# One-year outcomes of microhook trabeculotomy versus suture trabeculotomy ab interno

**DOI:** 10.1007/s00417-021-05333-7

**Published:** 2021-08-02

**Authors:** Hiroshi Yokoyama, Masashi Takata, Fumi Gomi

**Affiliations:** Department of Ophthalmology, Hyogo College of Medicine, 1-1 Mukogawa-cho, Nishinomiya, Hyogo 663-8501 Japan

**Keywords:** Gonioscopy-assisted suture trabeculotomy, Microhook trabeculotomy, Trabeculotomy ab interno, Goniotomy, MIGS

## Abstract

**Purpose:**

To compare clinical success rates and reductions in intraocular pressure (IOP) and IOP-lowering medication use following suture trabeculotomy ab interno (S group) or microhook trabeculotomy (μ group).

**Methods:**

This retrospective review collected data from S (*n* = 104, 122 eyes) and μ (*n* = 42, 47 eyes) groups who underwent treatment between June 1, 2016, and October 31, 2019, and had 12-month follow-up data including IOP, glaucoma medications, complications, and additional IOP-lowering procedures. The Kaplan–Meier survival analysis was used to evaluate treatment success rates defined as normal IOP (> 5 to ≤ 18 mm Hg), ≥ 20% reduction of IOP from baseline at two consecutive visits, and no further glaucoma surgery.

**Results:**

Schlemm’s canal opening was longer in the S group than in the μ group (*P* < 0.0001). The Kaplan–Meier survival analysis of all eyes showed cumulative clinical success rates in S and µ groups were 71.1% and 61.7% (*P* = 0.230). The Kaplan–Meier survival analysis of eyes with preoperative IOP ≥ 21 mmHg showed cumulative clinical success rates in S and μ groups were 80.4% and 60.0% (*P* = 0.0192). There were no significant differences in postoperative IOP at 1, 3, and 6 months (S group, 14.9 ± 5.6, 14.6 ± 4.5, 14.6 ± 3.9 mmHg; μ group, 15.8 ± 5.9, 15.2 ± 4.4, 14.7 ± 3.7 mmHg; *P* = 0.364, 0.443, 0.823), but postoperative IOP was significantly lower in the S group at 12 months (S group, 14.1 ± 3.1 mmHg; μ group, 15.6 ± 4.1 mmHg; *P* = 0.0361). There were no significant differences in postoperative numbers of glaucoma medications at 1, 3, 6, and 12 months (S group, 1.8 ± 1.6, 1.8 ± 1.5, 2.0 ± 1.6, 1.8 ± 1.5; μ group, 2.0 ± 1.6, 2.0 ± 1.6, 2.1 ± 1.6, 2.2 ± 1.7; *P* = 0.699, 0.420, 0.737, 0.198).

**Conclusion:**

S and µ group eyes achieved IOP reduction, but μ group eyes had lower clinical success rates among patients with high preoperative IOP at 12 months.

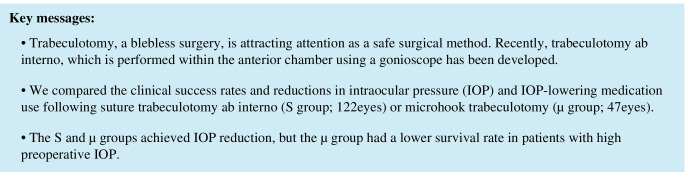

## Introduction

Glaucoma is a major vision-threatening disease worldwide, which is generally controlled by the reduction of intraocular pressure (IOP) [1]. When IOP-lowering eyedrops are ineffective, glaucoma surgery is considered. Trabeculectomy is a gold standard filtering surgery but hypotony immediately after the surgery is a serious adverse event. Trabeculectomy works well and it can maintain the visual field for a long time. In cases where trabeculectomy is not successful, glaucoma drainage implants (e.g., Ahmed valve and Baerveldt implant) are widely used. However, bacterial infection of the bleb and hypotony maculopathy are major serious adverse events related to these surgeries [2]. Accordingly, trabeculotomy (a blebless surgery) is receiving attention because of its potential for greater safety.

During trabeculotomy, the juxtacanalicular trabecular meshwork (TM) and inner wall of Schlemm’s canal (SC) are incised to reduce the resistance of aqueous fluid outflow. The procedure is performed with a metal probe inserted through the scleral flap, which comprises an ab externo approach [3]. Recently, trabeculotomy ab interno has been introduced, involving gonioscope use within the anterior chamber [4]. The advantages of the ab interno procedure are the absence of injury to the conjunctiva and sclera, reduced damage to Descemet’s membrane and the iris, and the creation of a wider incision in the TM. Suture trabeculotomy and microhook ab interno trabeculotomy have recently become common procedures in Japan. Suture trabeculotomy requires a complicated manipulation and considerable surgeon experience, but enables up to 360° incision [5]. The microhook trabeculotomy approach, introduced by Tanito et al. [6], is easier to perform within a shorter operation time, compared with suture trabeculotomy; microhook trabeculotomy incises up to approximately 240° (8 clock h) of the TM. A longer incision may accelerate the reduction in IOP, but might cause incision-associated hyphema or other side effects.

To the best of our knowledge, no studies have compared the efficacy and adverse events between suture trabeculotomy and microhook ab interno trabeculotomy. Here, we compared the treatment outcomes between suture trabeculotomy and microhook ab interno trabeculotomy.

## Patients and methods

This retrospective observational study was conducted at the Hyogo College of Medicine Hospital. The Institutional Review Board of Hyogo College of Medicine approved this study (No. 3535), which followed the tenets of the Declaration of Helsinki.

Patients were included if they had undergone suture trabeculotomy or microhook trabeculotomy at the Hyogo College of Medicine Hospital from June 1, 2016, to October 31, 2019, and had more than 12 months of follow-up. The exclusion criteria were as follows: prior glaucoma surgery, non-open-angle glaucoma (e.g., angle-closure glaucoma, pediatric glaucoma, and neovascular glaucoma), and corneal opacity that impaired angle visualization. The following data were extracted from medical records: age, sex, glaucoma type, lens status, surgical procedure (suture trabeculotomy ab interno or microhook trabeculotomy), presence or absence of simultaneous cataract surgery, range of SC incision, incidences of postoperative hyphema and transient IOP elevation, and requirement for additional glaucoma surgery.

Data regarding IOP and glaucoma drug scores were collected preoperatively and at 1 day, 1 week, and 1, 3, 6, and 12 months postoperatively. IOP was measured using a Goldmann applanation tonometer (Haag-Streit Diagnostics, Köniz, Switzerland). The glaucoma drug score was the number of glaucoma eye drop medications used by each patient: combination glaucoma drops were assigned 2 points whereas single glaucoma medications and oral carbonic anhydrase inhibitors were assigned 1 point. The extent of the SC incision was determined in 30° increments, up to a maximum of 360°, from surgical records. Postoperative hyphema was assessed by determining whether coagula- or niveau-forming hyphema in the anterior chamber persisted until 1 day or 1 week postoperatively. Transient postoperative IOP elevation was recorded as an IOP of ≥ 25 mmHg between 1 week and 3 months postoperatively, followed by a reduction to ≤ 21 mmHg.

### Surgical procedures

Suture trabeculotomy ab interno (S-LOT) was performed by a single surgeon (MT) and microhook trabeculotomy was performed by two surgeons (MT and HY). Both procedures were conducted as follows. Topical 4% lidocaine was applied preoperatively and standard sterile preparations were performed. After the induction of intracameral anesthesia using preservative-free 1% lidocaine, the anterior chamber was filled with an ophthalmic viscosurgical device (1% Healon®; AMO, Santa Ana, CA, USA). A surgical gonio lens (Volk, Mentor, OH, USA) was used to observe the TM. Approximately 30° of microscope tilt, combined with approximately 45° of head tilt, was required to achieve an optimal gonioscopic view of the angle. For patients aged > 50 years and/or those with a vision-reducing cataract, simultaneous phacoemulsification was performed with an additional 2.4-mm corneal incision.

S-LOT procedures were as follows, using a modified 360° suture trabeculotomy technique. First, a near-limbal corneal port was created on the temporal side using a 2.2-mm slit knife (Alcon, Fort Worth, TX, USA). Second, the SC was incised through a temporal corneal incision at an angle of 15° on the nasal side using a 20-gauge V-Lance Knife (Alcon). Third, the rounded tip of a 5–0 nylon suture was inserted into the SC using 25-gauge disposable forceps. Fourth, the suture was pulled through the same opening after it had been passed around the SC perimeter. When the suture could not advance further into SC, it was removed from the temporal corneal incision site to perform SC incision for the inserted range. Additionally, a suture was inserted into the opposite side of the SC incision site, then pulled through the tip of the 5–0 nylon suture. Finally, the surgeon attempted a 360° SC incision, as long and wide as possible.

Microhook trabeculotomy procedures were as follows. A temporal, nasal, upper corneal incision was created with a 20-gauge V-Lance Knife (Alcon). This incision was used to incise the SC, by means of the Tanito trabeculotomy microhook™ (Handaya, Tokyo, Japan), within the range of two to three quadrants.

### Outcome measures

The main outcome measure was clinical success, defined as follows: normal IOP (> 5 to ≤ 18 mmHg), ≥ 20% IOP reduction from baseline at two consecutive visits, and no requirement for further glaucoma surgery. The Kaplan–Meier survival analysis was used to evaluate the clinical success rates. Secondary measures were any differences in changes in the IOP and glaucoma drug scores, as well as the rates of adverse events and additional glaucoma surgeries, between the S and μ groups.

### Statistical analyses

The Mann–Whitney *U*-test or the Wilcoxon signed-rank test was used to assess group differences in continuous variables including age, follow-up duration, pre- and postoperative IOP values, glaucoma drug score, and SC opening range. Fisher’s exact test or Pearson’s *χ*^2^ test was used to assess group differences in categorical variables including sex, type of glaucoma, rate of simultaneous cataract surgery, and absence of transient IOP elevation. Analyses were performed with JMP Pro, version 14.0.0 (SAS Institute, Cary, NC, USA). Kaplan–Meier survival analyses were conducted to assess the long-term clinical success rates, which were compared using the log-rank test. For all analyses, *P* values and two-sided 95% confidence intervals were reported for point estimates. Statistical significance was defined as *P* < 0.05.

## Results

### Patient characteristics

Overall, 169 eyes from 146 patients were included in this study: 122 eyes from 104 patients who underwent suture trabeculotomy (S group) and 47 eyes of 42 patients who underwent microhook trabeculotomy (μ group). Patient characteristics are shown in Table 1. The mean age and sex did not significantly differ between the two groups. In addition, there were no significant differences in preoperative IOP, glaucoma drug score, glaucoma type, rate of lens absence, and rate of simultaneous cataract surgery between the two groups. Preoperative IOP ≥ 21 mmHg was observed in 88 eyes (72.1%) in the S group and 35 eyes (74.5%) in the μ group. The extent of SC incision was significantly greater in the S group (336.9 ± 51.9°) than in the µ group (215.1 ± 32.7°; *P* < 0.0001).

**Table 1:** Patient characteristics

	Total	S group	µ group	*P* value
	145 patients (169 eyes)	104 patients (122 eyes)	42patients (47 eyes)	
Age (mean ± SD) years	69.2 ± 12.3	69.2 ± 11.9	69.1 ± 13.5	0.830
Sex (male), *n* (%)	75 (51.7)	50 (48.0)	26 (61.9)	0.183
Preoperative IOP, mean ± SD (mmHg)	27.6 ± 10.0	27.7 ± 10.0	27.3 ± 10.2	0.767
Preoperative IOP ≥ 21 mmHg, *n* (%)	123 (72.8)	88 (72.1)	35 (74.5)	0.848
Preoperative the number of glaucoma medications, mean ± SD	4.0 ± 1.1	4.0 ± 1.0	3.9 ± 1.2	0.873
Type of glaucoma				0.734
POAG, *n* (%)	88 (52.1)	64 (52.5)	24 (51.1)	
Exfoliative, *n* (%)	42 (24.9)	32 (26.2)	10 (21.3)	
Steroid-induced, *n* (%)	17 (10.1)	12 (9.8)	5 (10.6)	
Others, *n* (%)	22 (13.0)	14 (11.5)	8 (17.0)	
Lens status				0.622
Phakic, *n* (%)	118 (69.8)	87 (71.3)	31 (65.9)	
Pseudophakic, *n* (%)	51 (30.2)	35 (28.7)	16 (34.0)	
Combined cataract extraction, *n* (%)	99 (68.3)	72 (59.0)	27 (57.4)	0.991
Extent range of incision in SC, mean ± SD (degrees)	303.0 ± 72.3	336.9 ± 51.9	215.1 ± 32.7	** < *0.0001***

Bold italic font indicates statistical significance (*P* < 0.05)

S group, gonioscopy-assisted suture trabeculotomy; µ group, microhook trabeculotomy. *IOP*, intraocular pressure; *SD*, standard deviation; *POAG*, primary open-angle glaucoma; *SC*, Schlemm’s canal

### Kaplan–Meier survival analysis

**Fig. 1:**
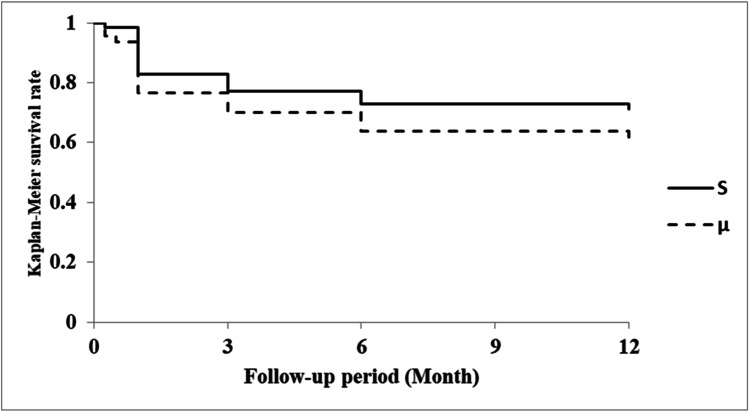
Kaplan–Meier curves for surgical success in all eyes, using the following criteria: normal IOP (> 5 to ≤ 18 mmHg), ≥ 20% IOP reduction from baseline at two consecutive visits, and no requirement for further glaucoma surgery. Lines representing suture trabeculotomy ab interno (—) and microhook trabeculotomy (–-) are shown for reference

**Fig. 2:**
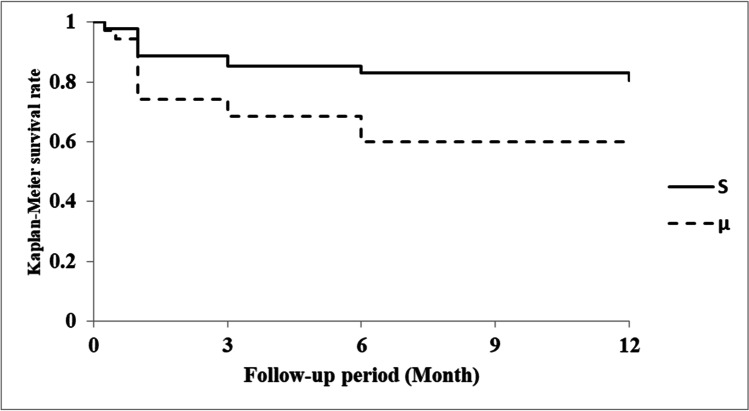
Kaplan–Meier curves for surgical success in eyes with preoperative IOP ≥ 21 mmHg, using the following criteria: normal IOP (> 5 to ≤ 18 mmHg), ≥ 20% IOP reduction from baseline at two consecutive visits, and no requirement for further glaucoma surgery. Lines representing suture trabeculotomy ab interno (—) and microhook trabeculotomy (–-) are shown for reference. S group, 88 eyes (72.1%); μ group, 35 eyes (74.5%)

Kaplan–Meier curves for all eyes are shown in Fig. 1. The Kaplan–Meier survival analysis indicated that the cumulative clinical success rates in the S and µ groups at 1 year postoperatively were 71.1% and 61.7%, respectively; these did not significantly differ between groups (log-rank test, *P* = 0.230). Among eyes with a preoperative IOP ≥ 21 mmHg, the cumulative clinical success rates in the S and µ groups at 1 year postoperatively were 80.4% and 60.0%, respectively (*P* = 0.0192; Fig. 2).

### Other outcomes

**Fig. 3:**
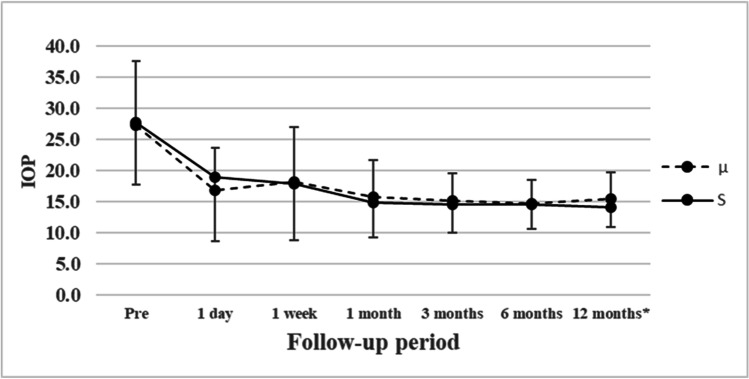
IOP for suture trabeculotomy ab interno (—) or microhook trabeculotomy (–-) in the 12-month follow-up period. *IOP at 12 months postoperatively was significantly lower in the S group than in the µ group (S group, 14.1 ± 3.1 mmHg; µ group, 15.6 ± 4.1 mmHg; *P* = 0.0361). Lines representing suture trabeculotomy ab interno (—) and microhook trabeculotomy (–-) are shown for reference

**Fig. 4:**
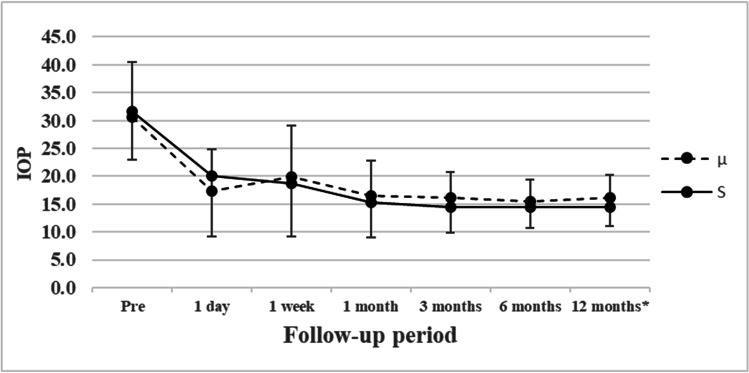
IOP for suture trabeculotomy ab interno (—) or microhook trabeculotomy (–-) in eyes with preoperative IOP ≥ 21 mmHg. *IOP at 12 months postoperatively was significantly lower in the S group than in the µ group (S group, 14.4 ± 3.4 mmHg; µ group, 16.1 ± 4.1 mmHg; *P* = 0.0438). Lines representing suture trabeculotomy ab interno (—) and microhook trabeculotomy (–-) are shown for reference. S group, 88 eyes (72.1%); μ group, 35 eyes (74.5%)

IOP significantly decreased after surgery in the S and µ groups (*P* < 0.00001 for all time points). In the S and μ groups, the postoperative IOP tended to be lower with combined phacoemulsification than with LOT alone. The postoperative IOP values for all eyes are shown in Fig. 3 and Table 2. For eyes with a preoperative IOP ≥ 21 mmHg, these values are shown in Fig. 4 and Table 3. Analysis of all eyes showed no significant differences in postoperative IOP from 1 day to 6 months postoperatively; at 12 months postoperatively, the IOP was significantly lower in the S group than in the µ group (S group, 14.1 ± 3.1 mmHg; µ group, 15.6 ± 4.1 mmHg; *P* = 0.0361). Analysis of eyes with a preoperative IOP ≥ 21 mmHg showed similar results; at 12 months postoperatively, the IOP was significantly lower in the S group than in the µ group (S group, 14.4 ± 3.4 mmHg; µ group, 16.1 ± 4.1 mmHg; *P* = 0.0438).

**Table 2:** Changes in IOP in all eyes

	Preoperative	1 day postoperative	1 week postoperative	1 month postoperative	3 months postoperative	6 months postoperative	12 months postoperative
S group, mean (median) ± SD (mmHg)	27.7 (25.5) ± 10.0 (*n* = 122)	18. 9 (15.5) ± 10.2 (*n* = 122)	18.0 (15) ± 9.1 (*n* = 121)	14.9 (14) ± 5.6 (*n* = 120)	14.6 (14) ± 4.5 (*n* = 116)	14.6 (14) ± 3.9 (*n* = 115)	14.1 (14) ± 3.1 (*n* = 102)
µ group, mean (median) ± SD (mmHg)	27.3 (24) ± 10.2 (*n* = 47)	16.9 (16) ± 6.8 (*n* = 47)	18.2 (14) ± 8.7 (*n* = 47)	15.8 (14) ± 5.9 (*n* = 46)	15.2 (14) ± 4.4 (*n* = 45)	14.7 (14) ± 3.7 (*n* = 43)	15.6 (16) ± 4.1 (*n* = 43)
*P* value	0.767	0.848	0.741	0.364	0.443	0.823	***0.0361***
S group with phacoemulsification, mean (median) ± SD (mmHg)	26.2 (25) ± 8.9 (*n* = 72)	20.2 (16.5) ± 10.6 (*n* = 72)	18.9 (16) ± 9.4 (*n* = 71)	13.7 (14) ± 2.9 (*n* = 71)	13.7 (14) ± 2.9 (*n* = 70)	14.1 (13.5) ± 3.3 (*n* = 70)	13.6 (14) ± 2.6 (*n* = 62)
µ group with phacoemulsification, mean (median) ± SD (mmHg)	22.8 (22) ± 7.1 (*n* = 27)	18.3 (16) ± 6.8 (*n* = 27)	18.7 (14) ± 9.5 (*n* = 27)	14.3 (14) ± 3.9 (*n* = 26)	14.0 (13) ± 4.2 (*n* = 26)	13.8 (14) ± 3.2 (*n* = 25)	14.6 (14) ± 3.9 (*n* = 25)
*P* value	0.0584	0.975	0.851	0.727	0.699	0.701	0.398
S group without phacoemulsification, mean (median) ± SD (mmHg)	29.8 (27) ± 11.1 (*n* = 50)	17.1 (14) ± 9.5 (*n* = 50)	16.7 (14) ± 8.6 (*n* = 50)	16.7 (15.0) ± 7.7 (*n* = 49)	15.9 (14.5) ± 6.1 (*n* = 46)	15.5 (16) ± 4.7 (*n* = 45)	14.9 (14.5) ± 3.7 (*n* = 40)
µ group without phacoemulsification, mean (median) ± SD (mmHg)	33.4 (31.5) ± 10.8 (*n* = 20)	15.0 (12.5) ± 6.5 (*n* = 20)	17.6 (14.5) ± 7.8 (*n* = 20)	17.8 (17.5) ± 7.4 (*n* = 20)	16.9 (17) ± 4.3 (*n* = 19)	16.1 (16.5) ± 4.0 (*n* = 18)	16.9 (17.5) ± 4.2 (*n* = 18)
*P* value	0.156	0.575	0.418	0.407	0.192	0.516	0.0602

Bold italic font indicates statistical significance (*P* < 0.05)

*SD*, standard deviation. S group, gonioscopy-assisted suture trabeculotomy; µ group, microhook trabeculotomy

**Table 3:** Changes in IOP in eyes with preoperative IOP ≥ 21 mmHg (S group, 88 eyes [72.1%]; μ group, 35 eyes [74.5%])

	Preoperative	1 day postoperative	1 week postoperative	1 month postoperative	3 months postoperative	6 months postoperative	12 months postoperative
S group, mean (median) ± SD (mmHg)	31.7 (30) ± 8.8 (*n* = 88)	20.0 (16) ± 10.8 (*n* = 87)	18.7 (16) ± 9.5 (*n* = 86)	15.3 (14) ± 6.3 (*n* = 85)	14.5 (14) ± 4.6 (*n* = 81)	14.4 (14) ± 3.8 (*n* = 80)	14.4 (14) ± 3.4 (*n* = 69)
µ group, mean (median) ± SD (mmHg)	30.6 (28) ± 9.8 (*n* = 35)	17.3 (17) ± 7.5 (*n* = 35)	19.9 (18) ± 9.2 (*n* = 35)	16.5 (14) ± 6.3 (*n* = 34)	16.2 (14) ± 4.5 (*n* = 33)	15.5 (15) ± 4.0 (*n* = 31)	16.1 (17) ± 4.1 (*n* = 31)
*P* value	0.331	0.546	0.316	0.276	0.0557	0.177	***0.0438***
S group with phacoemulsification, mean (median) ± SD (mmHg)	30.0 (30) ± 7.6 (*n* = 51)	21.3 (17) ± 11.3 (*n* = 51)	19.2 (16) ± 9.9 (*n* = 50)	13.8 (14) ± 3.0 (*n* = 50)	13.5 (13) ± 2.9 (*n* = 49)	14.0 (13) ± 3.6 (*n* = 49)	13.7 (14) ± 2.7 (*n* = 41)
µ group with phacoemulsification, mean (median) ± SD (mmHg)	26.2 (22) ± 7.4 (*n* = 16)	20.0 (19) ± 7.8 (*n* = 16)	22.2 (20) ± 10.3 (*n* = 16)	14.7 (14) ± 3.6 (*n* = 15)	15.3 (14) ± 4.6 (*n* = 15)	14.6 (14) ± 3.8 (*n* = 14)	15.0 (14) ± 3.8 (*n* = 14)
*P* value	***0.0197***	0.802	0.177	0.544	0.302	0.526	0.396
S group without phacoemulsification, mean(median) ± SD (mmHg)	34.0 (30) ± 9.9 (*n* = 37)	18.3 (14) ± 10.1 (*n* = 37)	18.1 (16) ± 9.1 (*n* = 37)	17.4 (15.5) ± 8.7 (*n* = 36)	15.9 (16.0) ± 6.1 (*n* = 33)	15.0 (14) ± 4.0 (*n* = 32)	15.4 (16) ± 4.1 (*n* = 28)
µ group without phacoemulsification, mean (median) ± SD (mmHg)	34.3 (33) ± 10.2 (*n* = 19)	15.1 (13) ± 6.6 (*n* = 19)	17.9 (15) ± 7.9 (*n* = 19)	17.8 (18.0) ± 7.6 (*n* = 19)	16.9 (17.5) ± 4.4 (*n* = 18)	16.2 (17) ± 4.1 (*n* = 17)	17.0 (18) ± 4.3 (*n* = 17)
*P* value	0.883	0.348	0.755	0.626	0.239	0.353	0.271

Bold italic font indicates statistical significance (*P* < 0.05)

*SD*, standard deviation. S group, gonioscopy-assisted suture trabeculotomy; µ group, microhook trabeculotomy

Postoperative glaucoma drug scores are shown in Table 4. The postoperative glaucoma drug score was significantly lower than the preoperative glaucoma drug score in the S and µ groups (*P* < 0.00001 for all time points). There were no significant differences in postoperative glaucoma drug score at any time point between the two groups. The proportions of glaucoma medication-free eyes are in Table 5. Table 5 shows the percentage of complete success: normal IOP (> 5 to ≤ 18 mmHg), ≥ 20% without any eye drops, and qualified success: normal IOP (> 5 to ≤ 18 mmHg), ≥ 20% with IOP-lowering eye drops. There were no significant differences in these proportions between the groups at 3, 6, and 12 months postoperatively.

**Table 4:** Changes in the number of glaucoma medications

	Preoperative	1 week postoperative	1 month postoperative	3 months postoperative	6 months postoperative	12 months postoperative
S group, mean (median) ± SD	4.0 (4) ± 1.0 (*n* = 122)	1.6 (1) ± 1.6 (*n* = 121)	1.8 (2) ± 1.6 (*n* = 120)	1.8 (2) ± 1.5 (*n* = 116)	2.0 (2) ± 1.6 (*n* = 115)	1.8 (2) ± 1.5 (*n* = 102)
µ group, mean (median) ± SD	3.9 (4) ± 1.2 (*n* = 47)	1.5 (2) ± 1.5 (*n* = 47)	2.0 (2) ± 1.6 (*n* = 46)	2.0 (2) ± 1.6 (*n* = 45)	2.1 (2) ± 1.6 (*n* = 43)	2.2 (2) ± 1.7 (*n* = 43)
*P* value	0.873	0.809	0.699	0.420	0.737	0.198

Bold italic font indicates statistical significance (*P* < 0.05)

*SD*, standard deviation. S group, gonioscopy-assisted suture trabeculotomy; µ group, microhook trabeculotomy

**Table 5:** Changes in proportion of glaucoma medication

	3 months postoperative	6 months postoperative	12 months postoperative
Complete success			
S group (%)	19.7 (24/122)	19.7 (24/122)	17.2 (21/122)
µ group (%)	19.1 (9/47)	19.1 (9/47)	19.1 (9/47)
* P* value	1.000	1.000	0.823
Qualified success			
S group (%)	52.5 (64/122)	49.2 (60/122)	50.0 (61/122)
µ group (%)	44.7 (21/47)	44.7 (21/47)	48.9 (23/47)
* P* value	0.394	0.611	1.000

Complete success: normal IOP (> 5 to ≤ 18 mmHg), ≥ 20% without any eye drops

Qualified success: normal IOP (> 5 to ≤ 18 mmHg), ≥ 20% with IOP-lowering eye drops

Bold italic font indicates statistical significance (*P *< 0.05)

S group, gonioscopy-assisted suture trabeculotomy; µ group, microhook trabeculotomy

### Safety

Hyphema at 1 day postoperatively was observed in 72 eyes (59.0%) in the S group and 24 eyes (51.1%) in the μ group. At 1 week postoperatively, hyphema was observed in 56 eyes (45.9%) in the S group and 17 eyes (36.2%) in the μ group. The proportions of eyes with hyphema at 1 day and 1 week were not significantly different between the two groups. Because of uncontrolled IOP elevation, hyphema washout was conducted in seven eyes (5.7%) in the S group and one eye (2.1%) in the µ group (Table 6).

**Table 6:** Intraoperative and postoperative complications

	Total	S group	µ group	*P* value
	145 patients (169 eyes)	104 patients (122 eyes)	42patients (47 eyes)	
Hyphema, *n* (%)				
Day 1	96 (56.8)	72 (59.0)	24 (51.1)	0.446
Week 1	73 (43.2)	56 (45.9)	17 (36.2)	0.332
IOP spike (≥ 25 mmHg), *n* (%)	35 (20.7)	25 (20.4)	10 (21.3)	1.000
Hypotony (IOP < 5 mmHg), *n* (%)	0	0	0	NA
Wound leaks	0	0	0	NA
Anterior chamber washout, *n* (%)	8 (2.1)	7 (5.7)	1 (2.1)	0.438
Additional glaucoma surgery, *n* (%)	15 (8.9)	11 (9.0)	4 (8.5)	1.000

Bold italic font indicates statistical significance (*P *< 0.05)

S group, gonioscopy-assisted suture trabeculotomy; µ group, microhook trabeculotomy. *IOP*, intraocular pressure; *NA*, not applicable

Postoperative transient IOP elevation occurred in 25 eyes (20.4%) in the S group and 10 eyes (21.3%) in the μ group; the proportion of eyes with transient IOP elevation did not significantly differ between groups. Additional glaucoma surgery was performed in 11 eyes (9.0%) in the S group and four eyes (8.5%) in the μ group; this proportion did not significantly differ between groups (Table 6).

## Discussion

In this study, we compared the postoperative results of trabeculotomy ab interno between suture and microhook approaches. The clinical success rates at 12 months postoperatively were similar (71.1% in the S group and 61.7% in the μ group), although there was a tendency toward a greater clinical success rate in the S group. The Kaplan–Meier survival analysis in eyes with a preoperative IOP of ≥ 21 mmHg showed a significantly greater clinical success rate at 12 months postoperatively in the S group than in the μ group. At 12 months postoperatively, the IOP showed a significantly greater reduction in the S group than in the μ group, although postoperative drug scores and the proportion of drug-free eyes did not significantly differ between groups. These results indicate that suture trabeculotomy lowers IOP over a longer duration, especially in eyes with higher preoperative IOP, compared with microhook trabeculotomy.

The main differences between these two procedures were the extent of SC incision and the overall surgical method. Suture trabeculotomy ideally enables a 360° incision, although some eyes do not allow a complete 360° incision; the mean incision size of suture trabeculotomy was 336.9 ± 51.9° in this study. Regarding microhook trabeculotomy, the original procedure [7] involved an incision of > 180° (6 clock h, two quadrants); however, recent modifications involve smaller incisions to control postoperative transient IOP elevation [8]. In this study, microhook trabeculotomy was performed using the original method; thus, the mean extent of incision was 215.1 ± 32.7°.

Theoretically, a wider SC incision could achieve a greater IOP-lowering effect by providing a greater reduction of aqueous fluid outflow resistance. However, previous reports have shown no significant differences in postoperative outcomes according to the range of SC incision using suture and microhook trabeculotomy methods. Manabe et al. [9] reported that the extent of incision within 150° and 320° of suture trabeculotomy ab externo did not correlate with postoperative IOP reduction. In a prospective study that compared outcomes according to the extent and location of the incision among groups with a 360° incision, and an upper- and lower-180° incision, Sato et al. [10] found no differences in IOP reduction and number of medications at 1 year postoperatively. Similarly, Mori et al. [8] compared 1-year outcomes and early surgery-related complications between one-quadrant and two-quadrant microhook trabeculotomy methods; they reported that the 1-year postoperative IOP, drug score, and Kaplan–Meier survival findings did not significantly differ between the one-quadrant and two-quadrant methods.

Using enucleated human eyes, Rosenquist et al. [11] reported that a 120° SC incision had 85% effectiveness for IOP reduction compared with a 360° SC incision; moreover, additional effectiveness was presumed to be achieved with incisions > 120°. Thus, our better 1-year outcomes in eyes undergoing suture trabeculotomy may be independent of the extent of the incision.

Several studies have investigated post-trabeculotomy morphological changes using anterior-segment optical coherence tomography (OCT). Akagi et al. [12] observed ciliochoroidal detachment (CCD) after ab interno trabeculotomy with a microsurgical device hand piece. At 3 days postoperatively, 42% of eyes had CCD and 58% of eyes had no CCD; postoperative CCD was associated with a lower IOP. This suggests that a non-conventional outflow pathway might be activated by trabeculotomy. Sato et al. [13] found similar results after 360° suture trabeculotomy ab interno. They hypothesized that CCD occurred more frequently in eyes subjected to 360° suture trabeculotomy compared with eyes subjected to trabectome surgery, because of the wider SC incision. However, the results showed that the presence and extent of CCD were similar for both surgical procedures: CCD was observed in 47.7% of eyes at 7 days postoperatively. Early postoperative CCD presumably had no effect on subsequent IOP; therefore, it cannot explain later differences in the success rates between the S and μ groups.

During microhook surgery, intraoperative OCT visualized the trabeculotomy cleft. Ishida et al. [14] identified three incisional patterns (anterior-opening, middle-opening, and posterior-opening), according to the predominant locations of TM flaps. The anterior-opening pattern was reportedly predominant. To the best of our knowledge, there are no data regarding the relationship between postoperative IOP and incisional pattern. Sato et al. [13] reported that they observed the root of the iris after intraoperative TM peeling during suture trabeculotomy suggesting S-LOT might form a continuous cyclodialysis cleft and posteriorly wider circumferential TM and SC incisions, leading to unconventional outflow. We hypothesize that this difference in cleft size partly explains the difference in 12-month outcomes between the two procedures.

A previous study [15] of microhook trabeculotomy demonstrated a poor prognosis in patients with higher preoperative IOP (e.g., those with exfoliative glaucoma). The collector channel distribution after SC differs between operated eyes and the collector channel and episcleral veins may become sclerotic under high IOP, resulting in a reduction in aqueous humor outflow function. In those eyes, a wider incision may be necessary to maintain outflow against the wound healing process. The patients in this study had undergone comparatively longer treatment at regular clinics before referral for surgery and this extended treatment might have affected the results. A recent report [16] suggested that atrophy of the collector channel system occurred depending on the degree of glaucoma. In patients with a short history of glaucoma medication, the collector channel function and subsequent function could be maintained with a more favorable surgical result.

Regarding surgical complications, a wider incision can have a greater risk of anterior chamber hemorrhage and transient IOP elevation [8]. However, there were no significant differences in such complications between the groups in the present study. Both procedures caused hyphema in almost half of the eyes and they caused a transient IOP spike in approximately 20% of eyes. Notably, the proportion of eyes that required anterior washout was higher in the S group, which generally causes unclear vision and high postoperative IOP.

There were several limitations in this study. First, the comparison was performed over a short duration (i.e., 12 months postoperatively) among a small number of participants. Second, because this was a retrospective study, postoperative IOP management was performed at the physician’s discretion, which might have affected the postoperative IOP and drug score. Third, the incisional extent of microhook trabeculotomy in our study was wider than that generally used in such surgeries, which may have influenced the results. However, we presume that it is important to identify the advantages and disadvantages of each surgical method, as the initial step of glaucomatous surgery.

In conclusion, the S and μ groups achieved an IOP reduction with a long-range SC incision while preserving the conjunctiva and sclera. The Kaplan–Meier survival analysis indicated that the cumulative clinical success rate did not significantly differ between the two groups, although eyes with high preoperative IOP had a greater clinical success rate in the S group than in the µ group. The optimal methods of trabeculotomy, including the extent of incision, require further investigation.

## Data Availability

Available.

## References

[1] Cvenkel B, Kolko M (2020). Current medical therapy and future trends in the management of glaucoma treatment. J Ophthalmol.

[2] HaiBo T, Xin K, ShiHeng L, Lin L (2015). Comparison of Ahmed glaucoma valve implantation and trabeculectomy for glaucoma: a systematic review and meta-analysis. PLoS ONE.

[3] Tanihara H, Negi A, Akimoto M, Terauchi H, Okudaira A, Kozaki J, Takeuchi A, Nagata M (1993). Surgical effects of trabeculotomy ab externo on adult eyes with primary open angle glaucoma and pseudoexfoliation syndrome. Arch Ophthalmol.

[4] Grover DS, Godfrey DG, Smith O, Feuer WJ, Montes de Oca I, Fellman RL (2014). Gonioscopy-assisted transluminal trabeculotomy, ab interno trabeculotomy: technique report and preliminary results. Ophthalmology.

[5] Sato T, Hirata A, Mizoguchi T (2015). Prospective, noncomparative, nonrandomized case study of short-term outcomes of 360 degrees suture trabeculotomy ab interno in patients with open-angle glaucoma. Clin Ophthalmol.

[6] Tanito M, Sano I, Ikeda Y, Fujihara E (2017). Short-term results of microhook ab interno trabeculotomy, a novel minimally invasive glaucoma surgery in Japanese eyes: initial case series. Acta Ophthalmol.

[7] Tanito M (2018). Microhook ab interno trabeculotomy, a novel minimally invasive glaucoma surgery. Clin Ophthalmol.

[8] Mori S, Murai Y, Ueda K, Sakamoto M, Kurimoto T, Yamada-Nakanishi Y, Nakamura M (2021). Comparison of efficacy and early surgery-related complications between one-quadrant and two-quadrant microhook ab interno trabeculotomy: a propensity score matched study. Acta Ophthalmol.

[9] Manabe SI, Sawaguchi S, Hayashi K (2017). The effect of the extent of the incision in the Schlemm canal on the surgical outcomes of suture trabeculotomy for open-angle glaucoma. Jpn J Ophthalmol.

[10] Sato T, Kawaji T (2020). 12-month randomised trial of 360° and 180° Schlemm’s canal incisions in suture trabeculotomy ab interno for open-angle glaucoma. Br J Ophthalmol.

[11] Rosenquist R, Epstein D, Melamed S, Johnson M, Grant WM (1989). Outflow resistance of enucleated human eyes at two different perfusion pressures and different extents of trabeculotomy. Curr Eye Res.

[12] Akagi T, Nakano E, Nakanishi H, Uji A, Yoshimura N (2016). Transient ciliochoroidal detachment after ab interno trabeculotomy for open-angle glaucoma: a prospective anterior-segment optical coherence tomography study. JAMA Ophthalmol.

[13] Sato T, Kawaji T, Hirata A (2019). Transient ciliochoroidal detachment after 360-degree suture trabeculotomy ab interno for open-angle glaucoma: 12-month follow-up. Eye (Lond).

[14] Ishida A, Sugihara K, Shirakami T, Tsutsui A, Manabe K, Tanito M (2020). Observation of gonio structures during microhook ab interno trabeculotomy using a novel digital microscope with integrated intraoperative optical coherence tomography. J Ophthalmol.

[15] Tanito M, Sugihara K, Tsutsui A, Hara K, Manabe K, Matsuoka Y (2021) Midterm results of microhook ab interno trabeculotomy in initial 560 eyes with glaucoma. J Clin Med 10(4). 10.3390/jcm10040814 10.3390/jcm10040814 PMC7922585 33671386

[16] Carreon T, van der Merwe E, Fellman RL, Johnstone M, Bhattacharya SK (2017). Aqueous outflow - a continuum from trabecular meshwork to episcleral veins. Prog Retin Eye Res.

